# The status of dental sleep medicine education in Australia and New Zealand in 2024

**DOI:** 10.1111/adj.13055

**Published:** 2024-12-30

**Authors:** L Tiwari, A Gikas, R Balasubramaniam

**Affiliations:** ^1^ UWA Dental School The University of Western Australia Nedlands Western Australia Australia; ^2^ Melbourne Teaching Health Clinics The University of Melbourne Carlton Victoria Australia; ^3^ Dental Unit The Alfred Hospital Melbourne Victoria Australia; ^4^ Dental and Maxillofacial Department Perth Children's Hospital Nedlands Western Australia Australia

**Keywords:** Competency, dental education, dental sleep medicine, sleep disordered breathing, sleep medicine education

## Abstract

**Background:**

This study aimed to evaluate the current status of dental sleep medicine education across dental schools in Australia and New Zealand and gain further insights into the educational background of dentists who have sat the Australasian Sleep Association Fellow of Dental Sleep Medicine examination in 2023.

**Methods:**

Online surveys were carried out, and descriptive statistics were used to analyse data.

**Results:**

All dental schools responded to the survey. Seventy per cent of the schools included dental sleep medicine as part of their curriculum, with an average total teaching time of 2.6 h. Dentists who sat the Fellow of dental sleep medicine examination spent on average 87 h preparing for the examination. All dental schools included discussion on pathophysiology of obstructive sleep apnoea and oral appliance therapy, but did not adequately discuss advanced sleep medicine topics, clinical aspects in treatment planning or contemporary dental sleep medicine topics, whereas dentists that completed the Fellow of dental sleep medicine examination gained knowledge in all aspects of the field.

**Conclusion:**

Findings from the study reveal that dental schools across Australia and New Zealand are not delivering adequate levels of education in dental sleep medicine, and hence the current dental sleep medicine curriculum needs to be reviewed and improved.

**Clinical Relevance**

The current study evaluated the status of dental sleep medicine education across dental schools in Australia and New Zealand and understand the educational background of dentists who completed the Australasian Sleep Association Fellow of dental sleep medicine examination. Results demonstrated that dental schools are lacking in providing foundational knowledge in dental sleep medicine to dental graduates, especially when compared to the education attained by those who sit the Fellow of dental sleep medicine examination. Results from this study reveal an urgent need to review the current dental sleep medicine curriculum being taught within dental schools in Australia and New Zealand.

## INTRODUCTION

Dental sleep medicine is concerned with the study of the oral and maxillofacial causes and consequences of sleep‐related problems such as sleep‐disordered breathing (snoring or obstructive sleep apnoea), sleep bruxism, orofacial pain and sleep‐related complaints, and to some extent gastro‐oesophageal reflux disease (GORD) and/or insomnia.^1^, ^2^ Obstructive sleep apnoea (OSA) is a key condition within dental sleep medicine. It is characterized by repetitive complete or partial closures of the upper airway, and is associated with snoring, daytime sleepiness, poor sleep quality and increased risks of cardiovascular diseases, stroke and decreased overall quality of life.^3^ Whilst traditionally, diagnosis and treatment of sleep‐disordered breathing fall under the umbrella of ‘medicine’, the important role of dentists, oral medicine specialists, oral and maxillofacial surgeons and orthodontists in the assessment and management of select sleep disorders has now been well established.^4^, ^5^

It has been suggested that one of the main roles of dentists in sleep medicine are to screen patients of all ages for sleep‐disordered breathing, screen for suboptimal craniofacial growth and development in children, to refer sleep‐disordered breathing patients to an appropriate health professional when indicated, and to provide oral appliance therapy when recommended.^2^, ^6^, ^7^ Further, dentists have the responsibility to assess for concomitant disorders including sleep bruxism, temporomandibular disorders, insomnia, headaches or GORD.^2^ Unfortunately, expertise in the assessment and management of these conditions is still lacking within the dental discipline, and this has been attributed to limited education and training exposure.^2^ Many studies call for greater consideration for dental sleep medicine within the dental curriculum worldwide, as well as better postgraduate training for dentists who are interested in specializing within this field.^3^, ^8^

In Australia and New Zealand, there is currently no standardized training in the field of dental sleep medicine across dental schools.^9^ In 2014, Balasubramaniam *et al*. reported an average of only 4.5 total hours of teaching dental sleep medicine across all surveyed undergraduate dental schools across Australia and New Zealand, suggesting a mere exposure to the discipline rather than providing sound foundational knowledge.^9^ Further, 55% of sleep medicine education was didactic, and only one dental school provided a 1‐h hands‐on clinical workshop.^9^ The study further found that the dental curriculum focussed primarily on obstructive sleep apnoea, and that advanced sleep medicine topics were rarely discussed.^9^ It was recommended by the authors that dental schools take leadership in providing standards of care to reflect contemporary practice, and to protect patients. Additionally, any training outside of dental schools in dental sleep medicine is currently derived from courses offered by oral appliance manufacturers, scientific meetings and continuing education programmes.^9^, ^10^ Further, the Australian Dental Council (ADC), an independent body for accrediting dental education and training programmes in Australia and New Zealand, defines the complex combination of knowledge, understanding, skills and attitudes required of newly qualified dentists to be considered ‘competent’ to be able to care for the public, currently does not require competency in the field of dental sleep medicine.^9^

To address the discrepancies in competencies, the Australasian Sleep Association, which is the peak scientific body in Australia and New Zealand representing clinicians, scientists and researchers working in sleep health and sleep medicine, established standardized assessment objectives and launched a certification programme in dental sleep medicine in 2022. To successfully complete the programme, candidates must pass a 3‐h multiple choice examination based on key topics and learning outcomes, submit a logbook of 15 dental sleep medicine cases, submit three case reports, attend a sleep laboratory placement, and obtain two references from sleep physicians. Eligible dentists who complete the certification requirements would be awarded the postnominals ‘Fellow of Dental Sleep Medicine’ or ‘FDSM’. These fellows would be listed on the Australasian Sleep Association public website to provide transparency to medical colleagues and patients on ‘credentialled’ dentists within the field of dental sleep medicine.

Given these developing changes in training standards in Australia and New Zealand over the last 10 years, this study aimed to evaluate the current status of dental sleep medicine education across dental schools in Australia and New Zealand and gain further insights into the educational background of dentists who have sat the Australasian Sleep Association FDSM examination and/or completed the FDSM programme in 2023.

## MATERIALS AND METHODS

An online survey was carried out with Qualtrics^XM^ after approval by the Human Research Ethics Committee of the University of Western Australia (UWA) (Re: 2024/ET000203), across all 10 dental schools in Australia and New Zealand to assess the dental sleep medicine curriculum for the 2024 academic year. Participants of the survey included heads of all the dental schools in Australia and New Zealand. The questions were based on learning outcomes as determined by Australasian Sleep Association and by the questionnaire utilized in the study by Balasubramaniam *et al*., and gathered data on (1): Hours spent teaching sleep medicine, (2) Teaching methods, (3) Department(s) involved in teaching, (4) Topics discussed, (5) Diagnosis reviewed, (6) Therapies discussed, (7) History, examination and imaging for patient selection, (8) Aspects of oral appliance therapies discussed, and (9) Best practice principles (Appendix S1).^9^ A second online survey carried out with Qualtrics^XM^ was sent to dentists who have sat the FDSM examination and/or completed the FDSM programme in 2023. Participants were recruited through the Australasian Sleep Association. Objectives tested by the survey were as above, in addition to demographic information on participants' educational status and time spent studying sleep medicine (Appendix S2). Simple descriptive statistics were employed for data analysis and performed with Qualtrics^XM^.

## RESULTS

### Dental sleep medicine education in dental schools in Australia and New Zealand

**Fig. 1:**
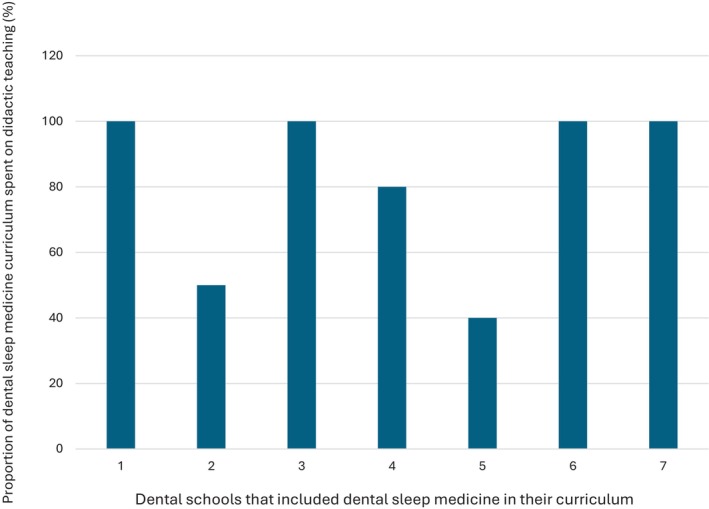
Proportion of time dental schools spent on didactic teaching in dental sleep medicine.

**Fig. 2:**
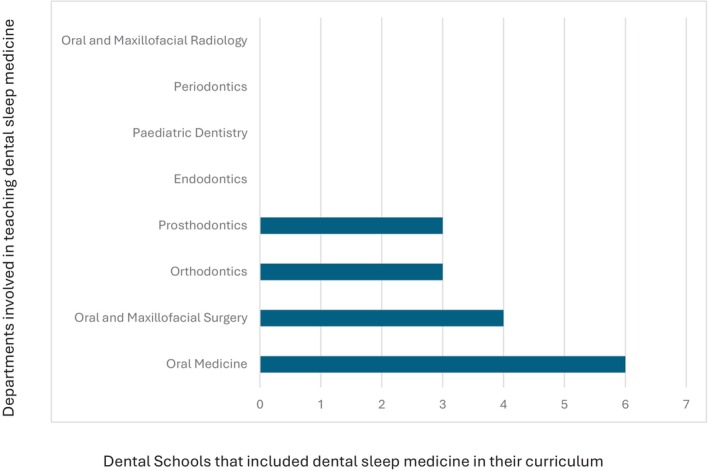
Departments involved in teaching dental sleep medicine in dental schools.

**Fig. 3:**
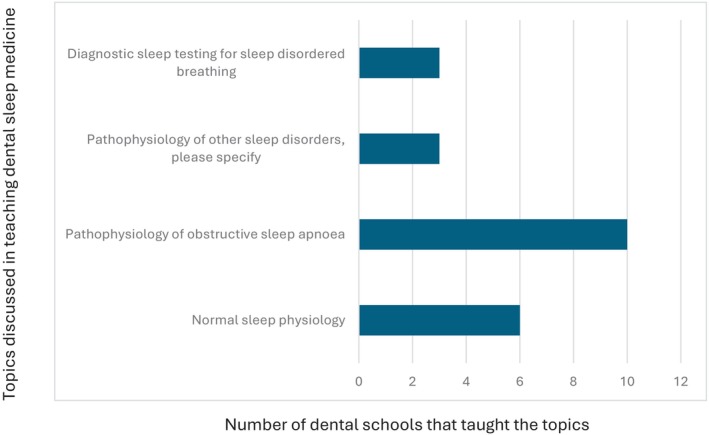
Topics discussed in the dental sleep medicine curriculum.

**Fig. 4:**
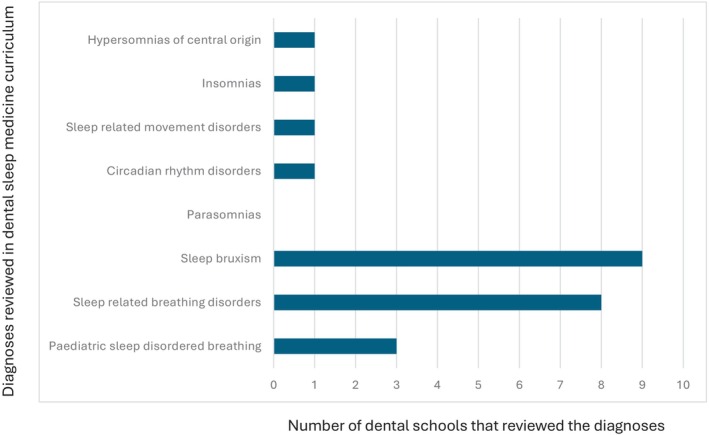
Diagnoses reviewed as part of the dental school curriculum.

**Fig. 5:**
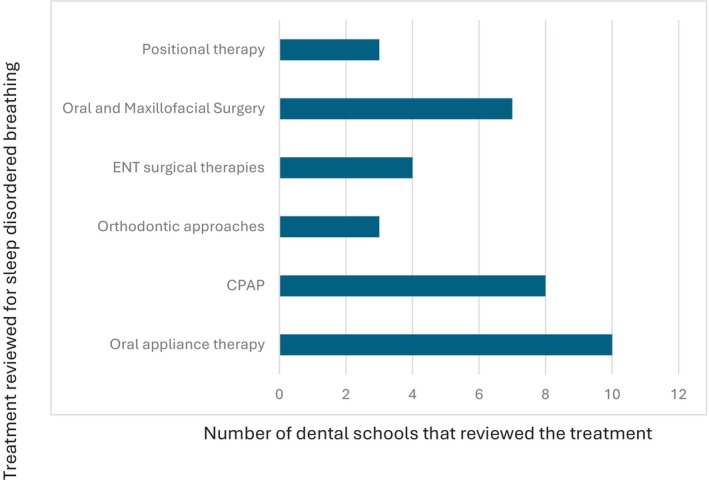
Discussion of treatment options for sleep‐disordered breathing within dental schools.

**Fig. 6:**
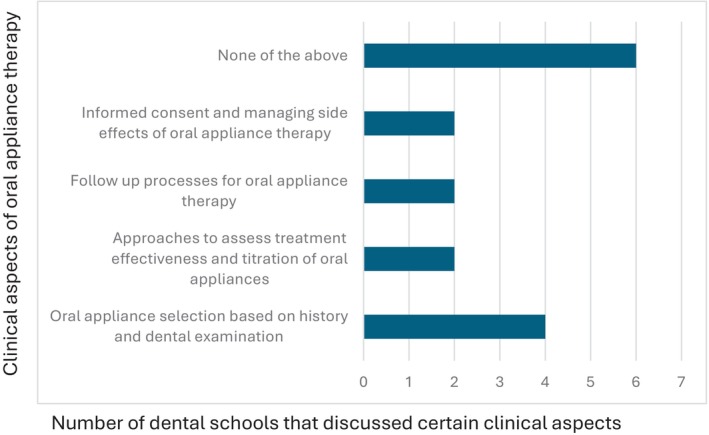
Discussion on clinical aspects of oral appliance therapy within dental schools.

**Fig. 7:**
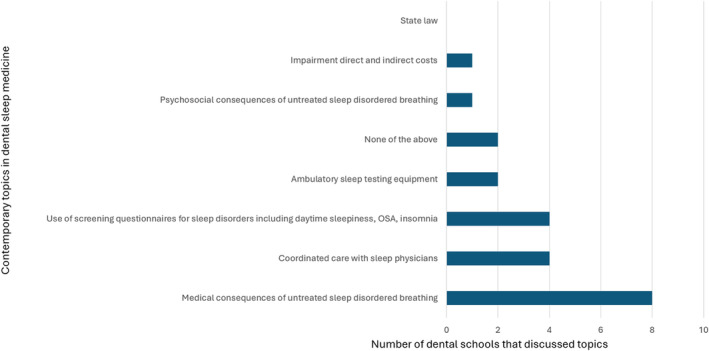
Discussion of contemporary topics in dental sleep medicine.

All 10 dental schools in Australia and New Zealand completed the survey. 40% of the schools award a Bachelor of Dental Surgery, 20% award a Bachelor of Dental Science, 20% award a Doctor of Dental Medicine and 20% award a Doctor of Dental Surgery. 70% of the schools included dental sleep medicine as part of their set dental curriculum, with most students receiving education in Year 3 (43%) and Year 4 (57%) of dental school. The average total teaching time was 2.6 h (0.5–6 h). Didactic teaching was the primary mode of delivery of education (Fig. 1). Only two schools provided clinical experience with the total proportion of time spent being 10% and 60%. One school spent 10% of the time reading academic articles. One school which does not have dental sleep medicine as part of their curriculum, included a discussion of the topic in other areas of interdisciplinary management, whilst the other two schools discussed the topic briefly in a lecture. The Oral Medicine speciality was most involved in teaching dental sleep medicine (Fig. 2). All dental schools included discussion on the pathophysiology of obstructive sleep apnoea (Fig. 3). Three schools also discussed pathophysiology of other sleep disorders, including sleep bruxism, insomnia, circadian rhythm disorders and restless legs syndrome. Sleep bruxism and sleep‐disordered breathing were the most frequent diagnoses reviewed in the curriculum (Fig. 4). Oral appliance therapy was discussed as a treatment option in all schools (Fig. 5), although the majority of the schools (60%) did not discuss the history and dental examination, approaches to assess effectiveness and titration of oral appliances, follow up process of oral appliance therapy and informed consent of oral appliance therapy (Fig. 6). Only four schools included discussion of clinical components focussing on dental sleep medicine history recording, treatment planning for sleep‐disordered breathing, and examination and imaging for patient selection. Eight dental schools discussed the medical consequences of untreated sleep‐disordered breathing (Fig. 7).

### Educational background of dentists who sat the Australasian Sleep Association Fellow of Dental Sleep Medicine Examination in 2023 and/or completed the Fellow of Dental Sleep Medicine

By the end of 2023, a total of 38 dentists had completed the FDSM examination. Of these, 27 participated in the survey resulting in a 71% response rate. Twenty six per cent of the respondents had completed the entire FDSM process, including case submissions. Among the respondents, 40% held the highest qualification of a bachelor's degree in dentistry. Additionally, those with further academic qualifications included two with a Bachelor of Science, four with a Master's degree, three with a Graduate Diploma, one with a Doctor of Clinical Dentistry, and one with a PhD. Furthermore, four held a Fellowship, and three had a Membership from the Royal Australasian College of Dental Surgeons (FRACDS, MRACDS).

The primary dental qualification was obtained as early as 1973 and as recently as 2020, with 39% graduating before 1995, 39% between 2005 and 2012, and 17% in the last decade. Notably, 92% of respondents reported that dental sleep medicine training was not included in their dental school curriculum, with only two receiving any dental sleep medicine training through the oral medicine department via didactic teaching.

Post‐graduation, the most common methods for obtaining dental sleep medicine training were industry‐sponsored continuing professional development courses (57%), Australasian Sleep Association courses and meetings (47%), other professional association meetings (33%), and formal university postgraduate degrees (33%). On average, respondents spent 87 h studying for the FDSM examination, with a range of 0–300 h.

**Fig. 8:**
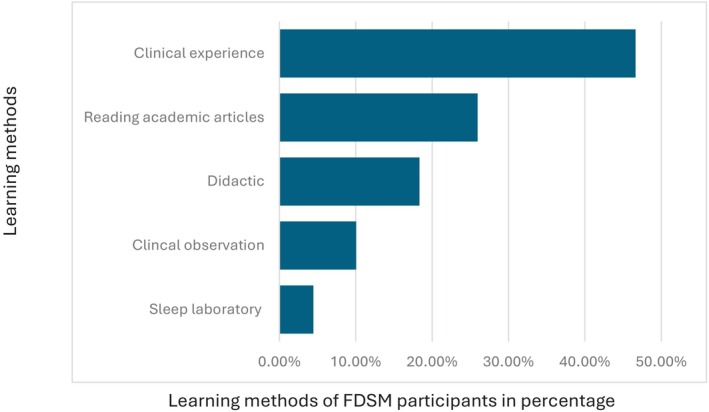
Methods of learning for the FDSM examination.

**Fig. 9:**
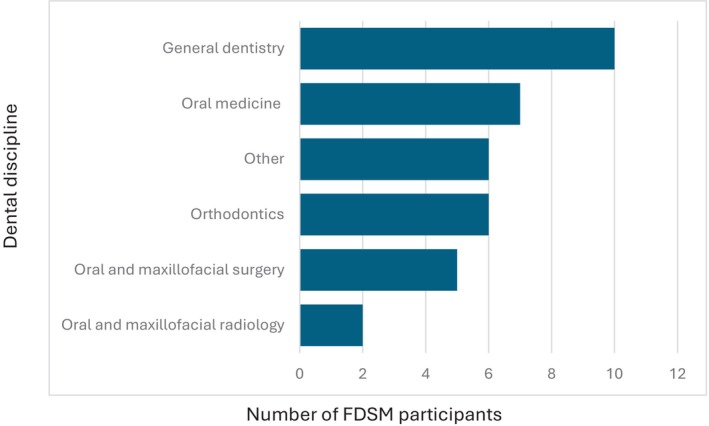
Dental disciplines involved in teaching dental sleep medicine to FDSM participants.

Regarding dental sleep medicine training methods, clinical experience was the most popular at 46.6%, followed by reading academic articles at 26%, didactic lectures at 18.35%, clinical observation (mentorship) at 10.5%, and sleep laboratory observations at 4.5% (Fig. 8). Among the sources of dental sleep medicine training, 77% were from general dentists, 54% from oral medicine specialists, 46% from orthodontists, 38% from oral and maxillofacial surgeons, and 15% from oral and maxillofacial radiologists. Additionally, 46% received some training from non‐dental practitioners (Fig. 9).

All respondents reported obtaining knowledge on dental sleep medicine topics of sleep physiology, pathophysiology of obstructive sleep apnoea and other sleep disorders, and diagnostic sleep testing for sleep‐disordered breathing. Notably, none of these topics were discussed during dental school, with the majority of respondents gaining knowledge in these areas through continuing professional development and courses after graduation or continuing professional development and courses in preparation for the FDSM examination (Table 1). Furthermore, respondents primarily reported acquiring knowledge from continuing professional development courses after graduation, and whilst in preparation for the FDSM examination for specific diagnoses and treatment options in dental sleep medicine (Table 2). Only two diagnoses were taught during dental school for a smaller proportion of respondents; sleep‐disordered breathing (4%), and sleep bruxism (22%). Further, only oral appliance therapy (4%), and orthodontic approaches (9%) were taught to some respondents during dental school (Table 3). The majority of respondents acquired knowledge of clinical components through continuing professional development courses after graduation or whilst in preparation for the FDSM examination (Table 4). 17% of the respondents also acquired knowledge through self‐guided study, a Graduate Diploma in Sleep Science, working as a respiratory sleep scientist or whilst working in a sleep clinic in conjunction with sleep physicians. The highest proportion of respondents with a range of 70%–78%, gained knowledge on aspects of oral appliance therapy through continuing professional development courses after graduation and before FDSM enrolment (Table 5). Most respondents attained knowledge on contemporary topics in dental sleep medicine, with the exception of ‘impairment direct and indirect costs’ (9%), ‘state law’ (22%) and ‘ambulatory sleep testing equipment’ (4%) (Table 6).

**Table 1:** Proportion of FDSM participants acquiring knowledge on topics on dental sleep medicine

Topics included in dental sleep medicine training	Location of knowledge acquired	% of FDSM participants	Additional comments
Sleep physiology	During dental school	0%	
	Continuing professional development and courses after graduation and before FDSM enrolment	65%	
	Continuing professional development and courses in preparation for the FDSM examination	48%	
	During Doctor of Clinical Dentistry or equivalent specialist training programme	4%	Doctor of Clinical Dentistry in Oral Medicine programme
	Other	17%	Self‐guided study Graduate Diploma in Sleep Science and working as a respiratory sleep scientist
	Was not discussed	0%	
Pathophysiology of obstructive sleep apnoea	During dental school	0%	
	Continuing professional development and courses after graduation and before FDSM enrolment	74%	
	Continuing professional development and courses in preparation for the FDSM examination	52%	
	During Doctor of Clinical Dentistry or equivalent specialist training programme	4%	Doctor of Clinical Dentistry in Oral Medicine programme
	Other	17%	Self‐guided study Graduate Diploma in Sleep Science and working as a respiratory sleep scientist
	Was not discussed	0%	
Pathophysiology of other sleep disorders	During dental school	0%	
	Continuing professional development and courses after graduation and before FDSM enrolment	48%	
	Continuing professional development and courses in preparation for the FDSM examination	57%	
	During Doctor of Clinical Dentistry or equivalent specialist training programme	4%	Doctor of Clinical Dentistry in Oral Medicine programme
	Other	17%	Self‐guided study Graduate Diploma in Sleep Science and working as a respiratory sleep scientist
	Was not discussed	0%	
Diagnostic sleep testing for sleep‐disordered breathing	During dental school	0%	
	Continuing professional development and courses after graduation and before FDSM enrolment	57%	
	Continuing professional development and courses in preparation for the FDSM examination	57%	
	During Doctor of Clinical Dentistry or equivalent specialist training programme	4%	Doctor of Clinical Dentistry in Oral Medicine programme
	Other	22%	Self‐guided study Graduate Diploma in Sleep Science and working as a respiratory sleep scientist Whilst working at private practice and in conjunction with sleep physicians
	Was not discussed	0%	

**Table 2:** Proportion of FDSM participants acquiring knowledge on specific diagnoses in dental sleep medicine

Review of the following diagnoses	Location of knowledge acquired	% of FDSM participants	Additional comments
Paediatric sleep‐disordered breathing	During dental school	0%	
	Continuing professional development and courses after graduation and before FDSM enrolment	70%	
	Continuing professional development and courses in preparation for the FDSM examination	61%	
	During Doctor of Clinical Dentistry or equivalent specialist training programme	9%	Doctor of Clinical Dentistry in Oral Medicine programme
	Other	9%	Self‐guided study Graduate Diploma in Sleep Science and working as a respiratory sleep scientist
	Was not discussed	0%	
Sleep‐disordered breathing	During dental school	4%	
	Continuing professional development and courses after graduation and before FDSM enrolment	57%	
	Continuing professional development and courses in preparation for the FDSM examination	61%	
	During Doctor of Clinical Dentistry or equivalent specialist training programme	9%	Doctor of Clinical Dentistry in Oral Medicine programme
	Other	13%	Self‐guided study Graduate Diploma in Sleep Science and working as a respiratory sleep scientist Whilst working at private practice and in conjunction with sleep physicians
	Was not discussed	0%	
Sleep bruxism	During dental school	22%	
	Continuing professional development and courses after graduation and before FDSM enrolment	70%	
	Continuing professional development and courses in preparation for the FDSM examination	61%	
	During Doctor of Clinical Dentistry or equivalent specialist training programme	9%	Doctor of Clinical Dentistry in Oral Medicine programme
	Other	17%	Self‐guided study Graduate diploma in Sleep Science and working as a respiratory sleep scientist Whilst working at practice and in conjunction with sleep physicians Observing oral medicine specialist (mentor)
	Was not discussed	0%	
Parasomnias	During dental school	0%	
	Continuing professional development and courses after graduation and before FDSM enrolment	52%	
	Continuing professional development and courses in preparation for the FDSM examination	61%	
	During Doctor of Clinical Dentistry or equivalent specialist training programme	13%	Doctor of Clinical Dentistry in Oral Medicine programme
	Other	9%	Self‐guided study Whilst working at private practice and in conjunction with sleep physicians
	Was not discussed	0%	
Circadian rhythm disorders	During dental school	0%	
	Continuing professional development and courses after graduation and before FDSM enrolment	48%	
	Continuing professional development and courses in preparation for the FDSM examination	65%	
	During Doctor of Clinical Dentistry or equivalent specialist training programme	9%	Doctor of Clinical Dentistry in Oral Medicine programme
	Other	13%	Self‐guided study Graduate diploma in Sleep Science and working as a respiratory sleep scientist Whilst working at private practice and in conjunction with sleep physicians
	Was not discussed	0%	
Sleep‐disordered breathing	During dental school	0%	
	Continuing professional development and courses after graduation and before FDSM enrolment	52%	
	Continuing professional development and courses in preparation for the FDSM examination	57%	
	During Doctor of Clinical Dentistry or equivalent specialist training programme	9%	Doctor of Clinical Dentistry in Oral Medicine programme
	Other	17%	Self‐guided study Graduate Diploma in Sleep Science and working as a respiratory sleep scientist Whilst working at private practice and in conjunction with sleep physicians
	Was not discussed	0%	
Insomnias	During dental school	0%	
	Continuing professional development and courses after graduation and before FDSM enrolment	57%	
	Continuing professional development and courses in preparation for the FDSM examination	61%	
	During Doctor of Clinical Dentistry or equivalent specialist training programme	4%	Doctor of Clinical Dentistry in Oral Medicine programme
	Other	22%	Self‐guided study Graduate Diploma in Sleep Science and working as a respiratory sleep scientist Whilst working at private practice and in conjunction with sleep physicians
	Was not discussed	0%	
Hypersomnias of central origin	During dental school	0%	
	Continuing professional development and courses after graduation and before FDSM enrolment	43%	
	Continuing professional development and courses in preparation for the FDSM examination	61%	
	During Doctor of Clinical Dentistry or equivalent specialist training programme	9%	Doctor of Clinical Dentistry in Oral Medicine programme
	Other	13%	Self‐guided study Graduate Diploma in Sleep Science and working as a respiratory sleep scientist Whilst working at private practice and in conjunction with sleep physicians
	Was not discussed	4%	

**Table 3:** Proportion of FDSM participants acquiring knowledge on treatment options

Discussion of treatment options	Location of knowledge acquired	% of FDSM participants	Additional comments
Oral Appliance Therapy	During dental school	4%	
	Continuing professional development and courses after graduation and before FDSM enrolment	78%	
	Continuing professional development and courses in preparation for the FDSM examination	52%	
	During Doctor of Clinical Dentistry or equivalent specialist training programme	9%	Doctor of Clinical Dentistry in Oral Medicine programme
	Other	17%	Self‐guided study Graduate Diploma in Sleep Science and working as a respiratory sleep scientist Whilst working at private practice and in conjunction with sleep physicians
	Was not discussed	0%	
CPAP	During dental school	0%	
	Continuing professional development and courses after graduation and before FDSM enrolment	61%	
	Continuing professional development and courses in preparation for the FDSM examination	61%	
	During Doctor of Clinical Dentistry or equivalent specialist training programme	4%	Doctor of Clinical Dentistry in Oral Medicine programme
	Other	22%	Self‐guided study Graduate Diploma in Sleep Science and working as a respiratory sleep scientist Whilst working at private practice and in conjunction with sleep physicians
	Was not discussed	0%	
Orthodontic approaches	During dental school	9%	
	Continuing professional development and courses after graduation and before FDSM enrolment	73%	
	Continuing professional development and courses in preparation for the FDSM examination	55%	
	During Doctor of Clinical Dentistry or equivalent specialist training programme	5%	Doctor of Clinical Dentistry in Oral Medicine programme
	Other	14%	Self‐guided study Graduate Diploma in Sleep Science and working as a respiratory sleep scientist Whilst working at private practice and in conjunction with Sleep physicians Observing oral medicine specialist (mentor)
	Was not discussed	0%	
ENT Surgical therapies	During dental school	0%	
	Continuing professional development and courses after graduation and before FDSM enrolment	61%	
	Continuing professional development and courses in preparation for the FDSM examination	61%	
	During Doctor of Clinical Dentistry or equivalent specialist training programme	4%	Doctor of Clinical Dentistry in Oral Medicine programme
	Other	22%	Self‐guided study Graduate Diploma in Sleep Science and working as a respiratory sleep scientist Whilst working at private practice and in conjunction with sleep physicians Observing oral medicine specialist (mentor)
	Was not discussed	0%	
Oral and Maxillofacial Surgery	During dental school	0%	
	Continuing professional development and courses after graduation and before FDSM enrolment	74%	
	Continuing professional development and courses in preparation for the FDSM examination	61%	
	During Doctor of Clinical Dentistry or equivalent specialist training programme	4%	Doctor of Clinical Dentistry in Oral Medicine programme
	Other	13%	Self‐guided study Graduate Diploma in Sleep Science and working as a respiratory sleep scientist Whilst working at private practice and in conjunction with sleep physicians
	Was not discussed	0%	
Positional therapy	During dental school	0%	
	Continuing professional development and courses after graduation and before FDSM enrolment	70%	
	Continuing professional development and courses in preparation for the FDSM examination	57%	
	During Doctor of Clinical Dentistry or equivalent specialist training programme	4%	Doctor of Clinical Dentistry in Oral Medicine programme
	Other	22%	Self‐guided study Graduate Diploma in Sleep Science and working as a respiratory sleep scientist Whilst working at private practice and in conjunction with sleep physicians
	Was not discussed	0%	

**Table 4:** Proportion of FDSM participants acquiring knowledge on clinical components of Dental Sleep Medicine

Discussion of clinical components	Location of knowledge acquired	% of FDSM participants	Additional comments
Dental sleep medicine history recording	During dental school	0%	
	Continuing professional development and courses after graduation and before FDSM enrolment	61%	
	Continuing professional development and courses in preparation for the FDSM examination	52%	
	During Doctor of Clinical Dentistry or equivalent specialist training programme	9%	Doctor of Clinical Dentistry in Oral Medicine programme
	Other	17%	Self‐guided study Observing oral medicine specialist Whilst working at private practice and in conjunction with sleep physicians
	Was not discussed	0%	
Examination and imaging for patient selection	During dental school	0%	
	Continuing professional development and courses after graduation and before FDSM enrolment	65%	
	Continuing professional development and courses in preparation for the FDSM examination	52%	
	During Doctor of Clinical Dentistry or equivalent specialist training programme	9%	Doctor of Clinical Dentistry in Oral Medicine programme
	Other	17%	Self‐guided study Graduate Diploma in Sleep Science and working as a respiratory sleep scientist Whilst working at private practice an in conjunction with sleep physicians
	Was not discussed	0%	
Treatment planning for dental sleep disorders	During dental school	0%	
	Continuing professional development and courses after graduation and before FDSM enrolment	65%	
	Continuing professional development and courses in preparation for the FDSM examination	52%	
	During Doctor of Clinical Dentistry or equivalent specialist training programme	9%	Doctor of Clinical Dentistry in Oral Medicine programme
	Other	17%	Self‐guided study, Graduate Diploma in Sleep Science and working as a respiratory sleep scientist Observing oral medicine specialist Whilst working at private practice and in conjunction with sleep physicians
	Was not discussed	0%	

**Table 5:** Proportion of FDSM participants acquiring knowledge on oral appliance therapy

Aspects of oral appliance therapy discussed	Location of knowledge acquired	% of FDSM participants	Additional comments
Oral appliance selection based on history and dental examination	During dental school	0%	
	Continuing professional development and courses after graduation and before FDSM enrolment	74%	
	Continuing professional development and courses in preparation for the FDSM examination	48%	
	During Doctor of Clinical Dentistry or equivalent specialist training programme	9%	Doctor of Clinical Dentistry in Oral Medicine programme
	Other	17%	Self‐guided study American Board of Dental Sleep Medicine webinar Research, observing oral medicine specialist Whilst working at private practice and in conjunction with sleep physicians
	Was not discussed	0%	
Approaches to assess treatment effectiveness and titration of oral appliances	During dental school	0%	
	Continuing professional development and courses after graduation and before FDSM enrolment	70%	
	Continuing professional development and courses in preparation for the FDSM examination	57%	
	During Doctor of Clinical Dentistry or equivalent specialist training programme	9%	Doctor of Clinical Dentistry in Oral Medicine programme
	Other	17%	Sleep research work Graduate Diploma in Sleep Science and working as a respiratory sleep scientist Observing oral medicine specialist Whilst working at Private practice an in conjunction with sleep physicians
	Was not discussed	0%	
Follow up process of oral appliance therapy	During dental school	0%	
	Continuing professional development and courses after graduation and before FDSM enrolment	78%	
	Continuing professional development and courses in preparation for the FDSM examination	57%	
	During Doctor of Clinical Dentistry or equivalent specialist training programme	9%	Doctor of Clinical Dentistry in Oral Medicine programme
	Other	13%	Graduate Diploma in Sleep Science and working as a respiratory sleep scientist Whilst working at private practice an in conjunction with sleep physicians
	Was not discussed	0%	
Informed consent and managing side effects of oral appliance therapy	During dental school	0%	
	Continuing professional development and courses after graduation and before FDSM enrolment	78%	
	Continuing professional development and courses in preparation for the FDSM examination	57%	
	During Doctor of Clinical Dentistry or equivalent specialist training programme	9%	Doctor of Clinical Dentistry in Oral Medicine programme
	Other	13%	JDSM articles Research, clinical practice, observing and learning from oral medicine specialist Whilst working at private practice and in conjunction with sleep physicians
	Was not discussed	0%	

**Table 6:** Proportion of FDSM participants acquiring knowledge on contemporary topics in dental sleep medicine

Discussion of contemporary topics	Location of knowledge acquired	% of FDSM participants	Additional comments
Medical consequences of untreated sleep‐disordered breathing	During dental school	0%	
	Continuing professional development and courses after graduation and before FDSM enrolment	70%	
	Continuing professional development and courses in preparation for the FDSM examination	61%	
	During Doctor of Clinical Dentistry or equivalent specialist training programme	4%	Doctor of Clinical Dentistry in Oral Medicine programme
	Other	13%	Graduate Diploma in Sleep Science and working as a respiratory sleep scientist Whilst working at private practice and in conjunction with sleep physicians, ENT surgeons and general medical practitioners
	Was not discussed	0%	
Coordinated care with sleep physicians	During dental school	0%	
	Continuing professional development and courses after graduation and before FDSM enrolment	74%	
	Continuing professional development and courses in preparation for the FDSM examination	57%	
	During Doctor of Clinical Dentistry or equivalent specialist training programme	9%	Doctor of Clinical Dentistry in Oral Medicine programme
	Other	9%	Graduate Diploma in Sleep Science and working as a respiratory sleep scientist Observing oral medicine specialist Whilst working at private practice and in conjunction with sleep physicians
	Was not discussed	0%	
Psychosocial consequences of untreated sleep‐disordered breathing	During dental school	0%	
	Continuing professional development and courses after graduation and before FDSM enrolment	70%	
	Continuing professional development and courses in preparation for the FDSM examination	52%	
	During Doctor of Clinical Dentistry or equivalent specialist training programme	4%	Doctor of Clinical Dentistry in Oral Medicine programme
	Other	17%	Graduate Diploma in Sleep Science and working as a respiratory sleep scientist Observing oral medicine specialist Whilst working at private practice and in conjunction with sleep physicians
	Was not discussed	0%	
Impairment direct and indirect costs	During dental school	0%	
	Continuing professional development and courses after graduation and before FDSM enrolment	57%	
	Continuing professional development and courses in preparation for the FDSM examination	57%	
	During Doctor of Clinical Dentistry or equivalent specialist training programme	4%	Doctor of Clinical Dentistry in Oral Medicine programme
	Other	9%	Whilst working at private practice
	Was not discussed	9%	
State law	During dental school	0%	
	Continuing professional development and courses after graduation and before FDSM enrolment	35%	
	Continuing professional development and courses in preparation for the FDSM examination	48%	
	During Doctor of Clinical Dentistry or equivalent specialist training programme	4%	Doctor of Clinical Dentistry in Oral Medicine programme
	Other	9%	Whilst working at private practice
	Was not discussed	22%	
Ambulatory sleep testing equipment	During dental school	0%	
	Continuing professional development and courses after graduation and before FDSM enrolment	61%	
	Continuing professional development and courses in preparation for the FDSM examination	52%	
	During Doctor of Clinical Dentistry or equivalent specialist training programme	4%	Doctor of Clinical Dentistry in Oral Medicine programme
	Other	13%	Graduate Diploma in Sleep Science and working as a respiratory sleep scientist Whilst working at private practice and in conjunction with sleep physicians
	Was not discussed	4%	
Use of screening questionnaires for sleep disorders including daytime sleepiness, obstructive sleep apnoea, insomnia	During dental school	4%	
	courses after graduation and before FDSM enrolment	65%	
	Continuing professional development and courses in preparation for the FDSM examination	57%	
	During Doctor of Clinical Dentistry or equivalent specialist training programme	4%	Doctor of Clinical Dentistry in Oral Medicine programme
	Other	26%	Self‐guided study Graduate Diploma in Sleep Science and working as a respiratory sleep scientist Whilst working at private practice and in conjunction with sleep physicians
	Was not discussed	0%	

## DISCUSSION

The important role that dentists play in the diagnosis and management of sleep disorders has now been well established.^3^ However, literature across the world continues to highlight the existing problems in teaching dental sleep medicine in dental schools and the subsequent lack of developing competencies for dentists within the field, resulting in a lack of confidence in dentists practising dental sleep medicine, in turn placing patients at risk of inadequate and inappropriate care.^2^, ^8^, ^9^, ^11^ In the past, dental sleep medicine education in Australia and New Zealand has demonstrated only an ‘awareness’ of the discipline within dental schools, and have fallen short in developing foundational, screening and teaching competencies.^9^ Since then, attempts have been made to standardize dental sleep medicine training in Australia and New Zealand and provide transparency in competency through the development of the FDSM programme by the Australasian Sleep Association, the peak body in Sleep Medicine in Australia and New Zealand. To understand if there has been any progression in the teaching of dental sleep medicine in Australia and New Zealand since 2014, the current study surveyed dental schools in Australia and New Zealand regarding their curriculum in dental sleep medicine. Additionally, we surveyed the dentists who have sat the FDSM examination and/or completed the FDSM programme in 2023 in order to understand the educational background of dentists who have obtained further qualifications in dental sleep medicine.

Findings from this study revealed that only 70% of dental schools in Australia and New Zealand including dental sleep medicine as part of their set dental curriculum, compared to 67% of the responding dental schools in Australia and New Zealand in 2014, indicating a minimal increase in adoption of dental sleep medicine curriculum in Australia and New Zealand overtime.^9^ It should be noted that all dental schools responded to the current survey, compared to an 85.7% response rate for schools that had graduated a class in 2014, as such providing a more accurate insight into the current status of education.^9^ When compared to other parts of the world, the education status is again comparable, with 56.2% of dental schools in France and 80.8% of Japanese dental schools include dental sleep medicine in their dental curriculum.^8^, ^12^ Interestingly in Australian and New Zealand dental schools, the average total hours spent on teaching dental sleep medicine has also reduced from 2014 (2.6 h as opposed to 4.5 h) and is also less in comparison to other dental schools around the world which report an average of 5.6 h in France, and 3.92 h in the USA, with the exception of the Middle East that had an average total teaching hours of 1.2 h.^8^, ^11^, ^13^ Conversely, dentists who completed the FDSM examination and/or programme, spent an average of 87 h studying for the FDSM examination. Perhaps the reduced hours in teaching can be attributed to varying responses provided by the different heads of schools in 2024 compared to 2014; however, the key concern remains that the hours of teaching dental sleep medicine have not increased over time at any rate. Interestingly, similar issues in education are present within the field of medicine, with basic sleep education not being adequately included in training programmes for primary healthcare providers as well as other allied health professionals including pharmacists, psychologists and nurses.^14^ Whilst data are limited, Maeklim *et al*. reported that in 2013, medical students in Australia and New Zealand received only an average of 2.5 h of sleep education.^14^ Meaklim *et al*. proposed an action agenda to help establish a sleep education strategy for healthcare providers.^14^ Similarly, urgent action is required for dental sleep medicine curriculum in Australian and New Zealand dental schools, given the significant discrepancy between a number of hours of teaching dental sleep medicine to graduating dentists, compared to the amount of study required to be considered competent in dental sleep medicine in Australia and New Zealand.

Further, the current study found that only Year 3 and Year 4 dental students received dental sleep medicine training, whereas in 2014, 55% of dental sleep medicine training was provided to 5th years, followed by 3^rd^ and 4^th^ years (19%), and 7% to 1st years.^9^ The change in this proportion is likely due to dental schools providing a 5‐year course in 2014, whereas 40% of current dental schools provide a 4‐year post‐graduate dental degree. Interestingly, 92% of FDSM respondents reported not receiving any dental sleep medicine training whilst they were in dental school. It should however be noted that 78% of the FDSM respondents obtained their primary dental qualification prior to 2012. This might introduce some bias in respondents' answers of where they acquired education in dental sleep medicine (industry‐sponsored continuing professional development courses, Australasian Sleep Association courses and meetings, professional association meetings and formal university postgraduate degrees), as pre‐2012 dental sleep medicine was only briefly incorporated within the dental curriculum, in turn prompting dentists to seek further education outside of dental schools.

The current study also demonstrated that didactic training continues to be the primary mode of delivery of education in Australian and New Zealand dental schools.^9^ Interestingly, only two dental schools provided a clinical component in their dental sleep medicine curriculum, compared to the reported four dental schools providing clinical training in 2014.^9^ Nonetheless, didactic training appears to be consistent with other dental sleep medicine curriculum in dental schools around the world, with studies noting that 78.4% of dental schools in the USA also provide a didactic approach to their teaching.^11^ Meanwhile, FDSM dentists reported a variety of training methods, with clinical experience being the most popular (46.6%), followed by reading academic articles (26%) and didactic lectures and clinical observations being the least popular (18.4%, and 10.5%, respectively). Experts in dental sleep medicine have long been suggesting that emphasis should be placed on providing knowledge through didactic and clinical material, in addition to having adequate clinical sleep laboratory training, and courses with hands‐on experiences in order to prepare future dental practitioners within the field.^10^ Despite this, the current mode of delivery of dental sleep medicine has not significantly changed since 2014, although current natural trends in learning suggest a much‐needed shift towards clinical experience, supporting previous recommendations, further reinforcing the required change in the delivery of dental sleep medicine education in dental schools.

Among dental schools in Australia and New Zealand, delivery of dental sleep medicine primarily appears to be carried out by the oral medicine discipline, although oral and maxillofacial surgery, prosthodontics and orthodontics disciplines are also involved in teaching dental sleep medicine to some degree. These findings are consistent with 2014 data, with the oral medicine department being most involved in teaching dental sleep medicine at that time as well.^9^ These findings also appear to be consistent across other dental schools worldwide, with 66.6% of dental schools in France involving the temporomandibular disorders/orofacial pain department (which falls within the scope of oral medicine in Australia and New Zealand) being the most frequently involved in teaching dental sleep medicine, followed by the orthodontics department (33.3%).^8^ It is interesting to note that in our study, FDSM respondents received most of their training from general dentists (77%), followed by select specialities including oral medicine specialists, orthodontists, oral and maxillofacial surgeons and oral and maxillofacial radiologists. This might be attributed to the level of clinical experience of general dentists as well as the links to industry and dental continuing professional development providers. General dentists also have a broad scope of practice, are well positioned to collaborate with other healthcare providers, are directly accessible to patients with sleep concerns and are aware of the growing demand for dental sleep medicine therapies and continuing education. Whilst the current study did not explore how the various departments integrate in teaching dental sleep medicine, the current setup at dental schools and existing participation of various disciplines places dental schools in the perfect position to provide the required multidisciplinary teaching needs of dental sleep medicine to its students.^10^

Regarding topics covered within dental sleep medicine curriculum, all dental schools in Australia and New Zealand provided a discussion on obstructive sleep apnoea including the pathophysiology of obstructive sleep apnoea, whilst 90% of dental schools reviewed diagnoses of sleep bruxism. This is a notable improvement from 2014, where only 67% of dental schools discussed sleep bruxism.^9^ Review of other sleep disorders including hypersomnias of central origin, insomnia, sleep‐related movement disorders and circadian rhythm disorders were only discussed by one dental school (10%). This is a reduction in comparison to 2014, where 50% of dental schools reviewed sleep‐related movement disorders, and 17% discussed insomnia.^9^ Interestingly, similar findings were noted in French dental schools with only 11.1% of dental schools teaching insomnia, parasomnia and restless leg syndrome, although 100% of participating dental schools discussed sleep bruxism.^8^ In the Middle East, dental schools most frequently taught insomnia (60%) and sleep bruxism (60%), followed by sleep‐disordered breathing (50%), whilst only 40% taught obstructive sleep apnoea.^13^ In comparison, all FDSM dentists reported to obtain knowledge in pathophysiology of obstructive sleep apnoea and sleep bruxism as well as the above‐mentioned advanced sleep medicine topics. Only 4% of respondents did not receive knowledge on hypersomnias of central origin. The difference in knowledge obtained between FDSM dentists compared to the knowledge provided by dental schools in Australia and New Zealand currently is substantial and clearly demarcates the levels of knowledge at which a graduating dental student receives by the end of their dental degree, compared to a dentist practising dental sleep medicine at a competent level. Given that sleep bruxism is the most managed sleep disorder in dentistry, is encountered by almost all general dentists, and is often comorbid with other sleep disorders such as obstructive sleep apnoea, sleep‐related movement disorders and insomnia, equal importance needs to be provided to teaching these conditions to ensure graduating dental students are receiving a comprehensive understanding of these comorbid conditions.^3^

The current study found that oral appliance therapy was discussed as a treatment option in all dental schools, followed by CPAP therapy, being discussed in 80% of dental schools. It appears that the curriculum has not changed significantly since 2014, as discussion on other relevant treatment options including oral and maxillofacial surgery, ENT surgical therapies and orthodontic approaches, positional therapy as well as contemporary topics including the use of screening questionnaires in dental sleep medicine were not consistently taught across all schools.^9^ Further, teaching on the topic of oral appliance therapy appears superficial, as 60% of dental schools did not discuss important clinical aspects of treatment planning including history and dental examination, assessing efficacy and titration of oral appliances, follow‐up processes of oral appliance therapy and obtaining informed consent. These findings are similar to those reported by dental schools in France, USA and Japan.^8^, ^11^, ^12^ Alternatively, all FDSM respondents reported attaining knowledge in all aspects of available treatment options for obstructive sleep apnoea, and in all clinical aspects of oral appliance therapy. Again, the majority of respondents received this training at continuing professional development courses before FDSM enrolment or continuing professional development courses whilst in preparation for the FDSM examination, rather than at dental school. Additionally, some respondents gained their knowledge through a Doctor of Clinical Dentistry in Oral Medicine programme, Graduate Diploma in Sleep Science, or whilst working in a sleep clinic. It is well known that dentists play a crucial role in providing oral appliance therapy when recommended in the management of obstructive sleep apnoea. It appears that dental schools in Australia and New Zealand are still lacking in providing adequate knowledge and training in this area, potentially placing patients at risk.

It has now long been questioned whether dentists have the foundational education required to competently treat patients with sleep‐disordered breathing.^9^ The current status of foundational education amongst dental schools in Australia and New Zealand does not appear to have significantly improved since 2014, with results still suggesting a mere ‘awareness’ of the discipline. Experts recommend that dentists must understand the physiology of sleep disorders, the differences between various types of sleep disorders to better screen patients, medical consequences of obstructive sleep apnoea and associated comorbidities.^2^ Results from this study show that educational needs within dental sleep medicine across dental schools are in significant and urgent need for development, and that current dental graduates are unable to meet the necessary competencies to manage patients with sleep‐disordered breathing.^3^ Specifically, there needs to be a significant increase into the number of hours of teaching dental sleep medicine to dental students and delve further into the specific knowledge areas within the field. The lack of knowledge in graduating dentists is highlighted further by the responses of dentists sitting on the FDSM examination. Whilst the response rate of dentists in the survey was only 71%, the significant differences between the dental school curriculum and level of knowledge attained by dentists sitting the FDSM examination are insightful to dictate the need for change. Perhaps the push for change in dental school education in dental sleep medicine might need to be driven by policy and competency changes within the Australian Dental Council, which still currently does not include professional competencies in the field of dental sleep medicine. Regardless, universities set the standards in education and are responsible for ensuring that graduating dentists are capable of undertaking and fulfilling their roles in contemporary dental practice. The field of sleep medicine is relatively young but the focus on a multidisciplinary team of health professionals brings dentists into the fold and the question around what are the highest qualifications a dentist can or should attain in sleep medicine is a discussion more around scope and the specialties growth. Dentists are able to enrol in various Masters programmes in Sleep Medicine and theory be trained to the highest level of sleep medicine. With these opportunities available, the expectation of the dentist's involvement in the multidisciplinary team is likely to evolve. In the meantime, it is reassuring that the dental profession via their peak professional bodies such as the American Academy of Dental Sleep Medicine, European Academy of Dental Sleep Medicine, German Society of Dental Sleep Medicine, and the Australasian Sleep Association recognize the dire need for credentialling within the field of dental sleep medicine and provide avenues for dentists who wish to practice dental sleep medicine with greater confidence and competency.

## CONCLUSION

Dental schools across Australia and New Zealand are still not providing adequate levels of education in the field of dental sleep medicine. Compared to dentists considered competent in practising dental sleep medicine, the current graduating dental students have a significant disparity with regards to the education they receive. There is an urgent need for dental schools to increase the amount of time spent teaching dental sleep medicine, and to provide greater depth of knowledge in all areas of dental sleep medicine in order to ensure standards are met for the current needs in practising dental sleep medicine.

## AUTHOR CONTRIBUTIONS

**L Tiwari:** Writing – original draft; methodology; formal analysis; project administration; data curation; investigation. **A Gikas:** Investigation; writing – original draft; methodology; writing – review and editing; formal analysis; data curation; project administration. **R Balasubramaniam:** Conceptualization; investigation; writing – review and editing; project administration; data curation; methodology; validation; visualization.

## FUNDING INFORMATION

There was no funding provided for this study.

## CONFLICT OF INTEREST

The authors do not have any conflict of interest to disclose.

## ETHICS STATEMENT

Ethics approval was obtained by the Human Research Ethics Committee of the University of Western Australia (UWA) (Re: 2024/ET000203).

## Supporting information


Appendix S1

## Data Availability

The data that support the findings of this study are available from the corresponding author upon reasonable request.
