# Supplementary material for: The status of dental sleep medicine education in Australia and New Zealand in 2024
Source: Aust Dent J. 2024 Dec 30;69(Suppl 1):S121–37. doi: 10.1111/adj.13055 (PMC11967704; doi:10.1111/adj.13055)
Supplement: Supplementary file 1 — Appendix S1 [file ADJ-69-S121-s001.docx]

**Appendix 1**

Dental Sleep Medicine Education across Dental Schools in Australia and New Zealand Questionnaire

1. What type of dental program does your school provide?
   1. Bachelor of Dental Science
   2. Bachelor of Dental Health Science/Master of Dentistry
   3. Bachelor of Dental Surgery
   4. Doctor of Dental Medicine
   5. Doctor of Dental Surgery
2. Does the dental curriculum include Dental Sleep Medicine?
   1. Yes
   2. No
3. What years receive training in Dental Sleep Medicine? (Select those that apply)
   1. Year 1
   2. Year 2
   3. Year 3
   4. Year 4
   5. Year 5
   6. N/A
4. In total, how many hours is allocated to teaching Dental Sleep Medicine in the curriculum? ________
5. What is the mode of delivery of teaching?
   1. Didactic, ____ % proportion
   2. Laboratory experience, ________ % proportion
   3. Clinical experience, _______ % proportion
   4. Clinical observation, _______ % proportion
   5. Reading academic articles, ______ % proportion
6. Which Department(s) are involved in teaching Dental Sleep Medicine? (select those that apply)
   1. Prosthodontics
   2. Oral Medicine
   3. Orthodontics
   4. Endodontics
   5. Paediatrics
   6. Oral and Maxillofacial Surgery
   7. Periodontics
   8. Oral and Maxillofacial Radiology
7. Does the curriculum, include discussion of the following topics? (select all that apply)
   1. Normal sleep physiology
   2. Pathophysiology of obstructive sleep apnoea
   3. Pathophysiology of other sleep disorders, please specify ___________
   4. Diagnostic sleep testing for sleep disordered breathing
   5. None of the above
8. Does the curriculum include review of the following diagnoses? (select all that apply)
   1. Paediatric sleep disordered breathing
   2. Sleep related breathing disorders
   3. Sleep bruxism
   4. Parasomnias
   5. Circadian rhythm disorders
   6. Sleep related movement disorders
   7. Insomnias
   8. Hypersomnias of central origin
   9. Other
9. Does the curriculum include discussion of the following treatment options? (select all that apply)
   1. Oral appliance therapy
   2. CPAP
   3. Orthodontic approaches
   4. ENT surgical therapies
   5. Oral and Maxillofacial Surgery
   6. Positional therapy
   7. None of the above
10. Does the curriculum include discussion of the following clinical components? (select all that apply)
    1. Dental sleep medicine history recording
    2. Examination and imaging for patient selection
    3. Treatment planning for dental sleep disorders
    4. None of the above
11. Does the curriculum include discussion of the following aspects of oral appliance therapy? (select all that apply)
    1. Oral appliance selection based on history and dental examination
    2. Approaches to assess treatment effectiveness and titration of oral appliances
    3. Follow up processes for oral appliance therapy
    4. Informed consent and managing side effects of oral appliance therapy
    5. None of the above
12. Does the curriculum include discussion of contemporary topics in dental sleep medicine including:
    1. Medical consequences of untreated sleep disordered breathing
    2. Coordinated care with sleep physicians
    3. Psychosocial consequences of untreated sleep disordered breathing
    4. Impairment direct and indirect costs
    5. State law
    6. Ambulatory sleep testing equipment
    7. Use of screening questionnaires for sleep disorders including daytime sleepiness, OSA, insomnia
    8. None of the above

Thank you for completing the questionnaire.

**Appendix 2**

Dental Sleep Medicine Education in candidates that completed the Fellow of Dental Sleep Medicine in 2023 Questionnaire

1. What are your qualifications? ___________________________
2. Where and in what year did you attain your primary qualification in dentistry? __________________
3. Did you receive any Dental Sleep Medicine (DSM) training as an undergraduate dental student as part of your dental school curriculum?
   1. Yes
   2. No – (questionnaire goes to question 6)
4. What was the mode of delivery of teaching of Dental Sleep Medicine throughout dental school?
   1. Didactic, ____ % proportion
   2. Laboratory experience, ________ % proportion
   3. Clinical experience, _______ % proportion
   4. Clinical observation, _______ % proportion
   5. Reading academic articles ______% proportion
5. Which Department(s) were involved in teaching Dental Sleep Medicine to you at dental school? (select those that apply)
   1. Prosthodontics
   2. Oral Medicine
   3. Orthodontics
   4. Endodontics
   5. Paediatrics
   6. Oral and Maxillofacial Surgery
   7. Periodontics
   8. Oral and Maxillofacial Radiology
   9. None of the above
6. What prior learning did you undertake **after** graduation and **before** commencing the FDSM? Please list all relevant academic and CPD courses noting who provided the course and year of completion. ___________________________________________________________
7. What was the mode of teaching Dental Sleep Medicine to you at dental school?
   1. Didactic, ____ % proportion
   2. Laboratory experience, ________ % proportion
   3. Clinical experience, _______ % proportion
   4. Clinical observation, _______% proportion
   5. Reading academic articles ______% proportion
8. **Once enrolled in the FDSM,** in total, how many hours did you spend studying Dental Sleep Medicine in preparation for the exam? ________
9. What was your method for learning and training in Dental Sleep Medicine?
   1. Didactic, ____ % proportion
   2. Laboratory experience, ________ % proportion
   3. Clinical experience, _______ % proportion
   4. Clinical observation, _______ % proportion
   5. Reading the articles on the FDSM reading list, ______% proportion
   6. Mentorship, ____________% proportion
10. Which discipline of Dental Practitioners were involved in teaching Dental Sleep Medicine to you **post-graduation**? (select those that apply)
    1. Prosthodontists
    2. Oral Medicine specialists
    3. Orthodontists
    4. Endodontists
    5. Paediatric dentists
    6. Oral and Maxillofacial Surgeons
    7. Periodontists
    8. Oral and Maxillofacial Radiologists
    9. General Dentists
    10. Other
11. Did your overall learning and training in Dental Sleep Medicine include the following topics? (select all that apply) Where was this knowledge acquired?
    1. Normal sleep physiology
       1. During dental school
       2. CPD and courses after graduation and before FDSM enrolment
       3. CPD and courses in preparation for the FDSM exam
       4. During Doctor of Clinical Dentistry or equivalent specialist training program (please specify specialty_________________)
       5. Other (please specify _________________)
    2. Pathophysiology of obstructive sleep apnoea
       1. During dental school
       2. CPD and courses after graduation and before FDSM enrolment
       3. CPD and courses in preparation for the FDSM exam
       4. During Doctor of Clinical Dentistry or equivalent specialist training program (please specify specialty _________________)
       5. Other (please specify _________________)
    3. Pathophysiology of other sleep disorders, please specify ___________
       1. During dental school
       2. CPD and courses after graduation and before FDSM enrolment
       3. CPD and courses in preparation for the FDSM exam
       4. During Doctor of Clinical Dentistry or equivalent specialist training program (please specify specialty _________________)
       5. Other (please specify _________________)
    4. Diagnostic sleep testing for sleep disordered breathing
       1. During dental school
       2. CPD and courses after graduation and before FDSM enrolment
       3. CPD and courses in preparation for the FDSM exam
       4. During Doctor of Clinical Dentistry or equivalent specialist training program (please specify specialty_________________)
       5. Other (please specify _________________)
    5. None of the above
12. Did your overall learning and training in Dental Sleep Medicine include review of the following diagnoses? (select all that apply) Where was this knowledge acquired?
    1. Paediatric sleep disordered breathing
       1. During dental school
       2. CPD and courses after graduation and before FDSM enrolment
       3. CPD and courses in preparation for the FDSM exam
       4. During Doctor of Clinical Dentistry or equivalent specialist training program (please specify specialty_________________)
       5. Other (please specify _________________)
    2. Sleep related breathing disorders
       1. During dental school
       2. CPD and courses after graduation and before FDSM enrolment
       3. CPD and courses in preparation for the FDSM exam
       4. During Doctor of Clinical Dentistry or equivalent specialist training program (please specify specialty_________________)
       5. Other (please specify _________________)
    3. Sleep bruxism
       1. During dental school
       2. CPD and courses after graduation and before FDSM enrolment
       3. CPD and courses in preparation for the FDSM exam
       4. During Doctor of Clinical Dentistry or equivalent specialist training program (please specify specialty_________________)
       5. Other (please specify _________________)
    4. Parasomnias
       1. During dental school
       2. CPD and courses after graduation and before FDSM enrolment
       3. CPD and courses in preparation for the FDSM exam
       4. During Doctor of Clinical Dentistry or equivalent specialist training program (please specify specialty_________________)
       5. Other (please specify _________________)
    5. Circadian rhythm disorders
       1. During dental school
       2. CPD and courses after graduation and before FDSM enrolment
       3. CPD and courses in preparation for the FDSM exam
       4. During Doctor of Clinical Dentistry or equivalent specialist training program (please specify specialty_________________)
       5. Other (please specify _________________)
    6. Sleep related movement disorders
       1. During dental school
       2. CPD and courses after graduation and before FDSM enrolment
       3. CPD and courses in preparation for the FDSM exam
       4. During Doctor of Clinical Dentistry or equivalent specialist training program (please specify specialty_________________)
       5. Other (please specify _________________)
    7. Insomnias
       1. During dental school
       2. CPD and courses after graduation and before FDSM enrolment
       3. CPD and courses in preparation for the FDSM exam
       4. During Doctor of Clinical Dentistry or equivalent specialist training program (please specify specialty_________________)
       5. Other (please specify _________________)
    8. Hypersomnias of central origin
       1. During dental school
       2. Continuing professional development and courses after graduation and before FDSM enrolment
       3. Continuing professional development and courses in preparation for the FDSM exam
       4. During Doctor of Clinical Dentistry or equivalent specialist training program (please specify specialty_________________)
       5. Other (please specify _________________)
13. Did your overall learning and training in Dental Sleep Medicine include discussion of the following treatment options? (select all that apply) If yes, where was this knowledge acquired?
    1. Oral appliance therapy
       1. During dental school
       2. CPD and courses after graduation and before FDSM enrolment
       3. CPD and courses in preparation for the FDSM exam
       4. During Doctor of Clinical Dentistry or equivalent specialist training program (please specify specialty_________________)
       5. Other (please specify _________________)
    2. CPAP
       1. During dental school
       2. CPD and courses after graduation and before FDSM enrolment
       3. CPD and courses in preparation for the FDSM exam
       4. During Doctor of Clinical Dentistry or equivalent specialist training program (please specify specialty_________________)
       5. Other (please specify _________________)
    3. Orthodontic approaches
       1. During dental school
       2. CPD and courses after graduation and before FDSM enrolment
       3. CPD and courses in preparation for the FDSM exam
       4. During Doctor of Clinical Dentistry or equivalent specialist training program (please specify specialty_________________)
       5. Other (please specify _________________)
    4. ENT surgical therapies
       1. During dental school
       2. CPD and courses after graduation and before FDSM enrolment
       3. CPD and courses in preparation for the FDSM exam
       4. During Doctor of Clinical Dentistry or equivalent specialist training program (please specify specialty_________________)
       5. Other (please specify _________________)
    5. Oral and Maxillofacial Surgery
       1. During dental school
       2. CPD and courses after graduation and before FDSM enrolment
       3. CPD and courses in preparation for the FDSM exam
       4. During Doctor of Clinical Dentistry or equivalent specialist training program (please specify specialty_________________)
       5. Other (please specify _________________)
    6. Positional therapy
       1. During dental school
       2. CPD and courses after graduation and before FDSM enrolment
       3. CPD and courses in preparation for the FDSM exam
       4. During Doctor of Clinical Dentistry or equivalent specialist training program (please specify specialty_________________)
       5. Other (please specify _________________)
    7. None of the above
14. In your overall learning and training in Dental Sleep Medicine, which of the following clinical components were discussed? If yes, where was this knowledge acquired (select all that apply)
    1. Dental sleep medicine history recording
       1. During dental school
       2. CPD and courses after graduation and before FDSM enrolment
       3. CPD and courses in preparation for the FDSM exam
       4. During Doctor of Clinical Dentistry or equivalent specialist training program (please specify specialty_________________)
       5. Other (please specify _________________)
    2. Examination and imaging for patient selection
       1. During dental school
       2. CPD and courses after graduation and before FDSM enrolment
       3. CPD and courses in preparation for the FDSM exam
       4. During Doctor of Clinical Dentistry or equivalent specialist training program (please specify specialty_________________)
       5. Other (please specify _________________)
    3. Treatment planning for dental sleep disorders
       1. During dental school
       2. CPD and courses after graduation and before FDSM enrolment
       3. CPD and courses in preparation for the FDSM exam
       4. During Doctor of Clinical Dentistry or equivalent specialist training program (please specify specialty_________________)
       5. Other (please specify _________________)
    4. None of the above
15. In your overall learning and training in Dental Sleep Medicine, which of the following aspects of oral appliance therapy was discussed? If yes, where was this knowledge acquired? (select all that apply)
    1. Oral appliance selection based on history and dental examination
       1. During dental school
       2. CPD and courses after graduation and before FDSM enrolment
       3. CPD and courses in preparation for the FDSM exam
       4. During Doctor of Clinical Dentistry or equivalent specialist training program (please specify specialty_________________)
       5. Other (please specify _________________)
    2. Approaches to assess treatment effectiveness and titration of oral appliances
       1. During dental school
       2. CPD and courses after graduation and before FDSM enrolment
       3. CPD and courses in preparation for the FDSM exam
       4. During Doctor of Clinical Dentistry or equivalent specialist training program (please specify specialty_________________)
       5. Other (please specify _________________)
    3. Follow up processes for oral appliance therapy
       1. During dental school
       2. CPD and courses after graduation and before FDSM enrolment
       3. CPD and courses in preparation for the FDSM exam
       4. During Doctor of Clinical Dentistry or equivalent specialist training program (please specify specialty_________________)
       5. Other (please specify _________________)
    4. Informed consent and managing side effects of oral appliance therapy
       1. During dental school
       2. CPD and courses after graduation and before FDSM enrolment
       3. CPD and courses in preparation for the FDSM exam
       4. During Doctor of Clinical Dentistry or equivalent specialist training program (please specify specialty_________________)
       5. Other (please specify _________________)
    5. None of the above
16. Did your overall learning and training in Dental Sleep Medicine include discussion of contemporary topics? If yes, where was this knowledge acquired?:
    1. Medical consequences of untreated sleep disordered breathing
       1. During dental school
       2. CPD and courses after graduation and before FDSM enrolment
       3. CPD and courses in preparation for the FDSM exam
       4. During Doctor of Clinical Dentistry or equivalent specialist training program (please specify specialty_________________)
       5. Other (please specify _________________)
    2. Coordinated care with sleep physicians
       1. During dental school
       2. CPD and courses after graduation and before FDSM enrolment
       3. CPD and courses in preparation for the FDSM exam
       4. During Doctor of Clinical Dentistry or equivalent specialist training program (please specify specialty_________________)
       5. Other (please specify _________________)
    3. Psychosocial consequences of untreated sleep disordered breathing
       1. During dental school
       2. CPD and courses after graduation and before FDSM enrolment
       3. CPD and courses in preparation for the FDSM exam
       4. During Doctor of Clinical Dentistry or equivalent specialist training program (please specify specialty_________________)
       5. Other (please specify _________________)
    4. Impairment direct and indirect costs
       1. During dental school
       2. CPD and courses after graduation and before FDSM enrolment
       3. CPD and courses in preparation for the FDSM exam
       4. During Doctor of Clinical Dentistry or equivalent specialist training program (please specify specialty_________________)
       5. Other (please specify _________________)
    5. State law
       1. During dental school
       2. CPD and courses after graduation and before FDSM enrolment
       3. CPD and courses in preparation for the FDSM exam
       4. During Doctor of Clinical Dentistry or equivalent specialist training program (please specify specialty_________________)
       5. Other (please specify _________________)
    6. Ambulatory sleep testing equipment
       1. During dental school
       2. CPD and courses after graduation and before FDSM enrolment
       3. CPD and courses in preparation for the FDSM exam
       4. During Doctor of Clinical Dentistry or equivalent specialist training program (please specify specialty_________________)
       5. Other (please specify _________________)
    7. Use of screening questionnaires for sleep disorders including daytime sleepiness, OSA, insomnia
       1. During dental school
       2. CPD and courses after graduation and before FDSM enrolment
       3. CPD and courses in preparation for the FDSM exam
       4. During Doctor of Clinical Dentistry or equivalent specialist training program (please specify specialty_________________)
       5. Other (please specify _________________)
    8. None of the above

Thank you for completing the questionnaire.
